# Healthcare professionals’ knowledge, attitudes, and practices regarding perioperative ischemic stroke in elderly patients

**DOI:** 10.3389/fmed.2026.1745538

**Published:** 2026-10-09

**Authors:** Li Zhang, Hao Nie, Hui Sun, Jianwei Li, Yunpeng Hei, Maoxian Zhang

**Affiliations:** 1 People’s Hospital of Ningxia Hui Autonomous Region, Ningxia Medical University, Yinchuan, China; 2 Department of Anesthesiology, People’s Hospital of Ningxia Hui Autonomous Region, Yinchuan, China; 3 The Third Clinical Medical College of Ningxia Medical University, Yinchuan, China

**Keywords:** aged, cross-sectional studies, health knowledge, attitudes, and practice, perioperative care, stroke

## Abstract

**Objective:**

This study aimed to evaluate healthcare professionals’ knowledge, attitudes, and practices (KAP) related to perioperative ischemic stroke in elderly patients.

**Methods:**

A single-center, cross-sectional survey was conducted at a tertiary hospital in Ningxia, China, between 1 March 2025 and 1 June 2025. Healthcare professionals were recruited by convenience sampling and completed a structured questionnaire covering demographic information and their KAP regarding perioperative ischemic stroke.

**Results:**

A total of 273 valid responses were included in the final analysis. The majority of respondents were physicians (75.46%), with most affiliated with anesthesiology departments (86.08%). The scores for knowledge, attitude, and practice were 14.41 ± 4.22 (possible range: 0–20), 36.77 ± 3.57 (possible range: 8–40), and 30.07 ± 6.51 (possible range: 8–40), respectively. Structural equation modeling showed that knowledge, attitude, and practice were positively interrelated, whereas training related to perioperative ischemic stroke and specialization in elderly patient care showed negative associations with knowledge or practice (*β* range: −0.287 to 0.352; all *P* < 0.05). Knowledge was also indirectly associated with practice through attitude, whereas training and specialization in elderly care showed negative indirect associations with attitude and practice.

**Conclusion:**

Healthcare professionals exhibited inadequate perceived knowledge, positive attitudes, and suboptimal practices regarding perioperative ischemic stroke in elderly patients. These findings underscore the necessity for targeted, evidence-based educational interventions to strengthen knowledge awareness and practice-related competencies, thereby addressing the observed gaps between perceived knowledge, attitudes, and reported practices.

## Background

Perioperative ischemic stroke represents a devastating complication occurring during or immediately following surgical procedures, with elderly patients demonstrating disproportionately elevated susceptibility and experiencing substantial morbidity and mortality rates (1, 2). This heightened vulnerability stems from the increased prevalence of established cerebrovascular risk factors within this demographic, including atherosclerosis, atrial fibrillation, hypertension, and diabetes mellitus, which collectively amplify stroke susceptibility (3, 4). The convergence of expanding surgical volumes and global demographic aging has precipitated a concerning upward trajectory in perioperative stroke incidence.

Contemporary epidemiological data from the United States reveal perioperative stroke rates ranging from 0.32% to 0.42% following non-cardiac surgical procedures, with notably higher frequencies documented in specialized interventions such as neurosurgical and cardiovascular operations (approximately 1%–1.25%) compared with general surgical procedures (0.1%–1.0%) (5, 6). Comparable rates have also been reported in other developed countries, although direct cross-country comparison is limited by differences in surgical case-mix, stroke ascertainment (clinically apparent vs. imaging-detected), and follow-up windows. Population-based and registry studies in European nations such as the United Kingdom and Germany report perioperative ischemic stroke incidences of approximately 0.3%–0.6% following non-cardiac surgery, with higher rates after vascular and major orthopedic procedures. Similarly, cohort data from Japan and South Korea suggest an incidence of approximately 0.2%–0.5% after non-cardiac surgery, with rates rising in cardiac and neurosurgical operations (7). Furthermore, the clinical burden extends beyond clinically apparent events, as covert cerebral infarctions—detectable exclusively through postoperative magnetic resonance imaging—affect up to 7% of elderly patients undergoing non-cardiac surgery and correlate with increased risks of postoperative delirium and cognitive deterioration (8).

Within the Chinese healthcare context, the stroke burden continues its alarming ascent, with age-standardized ischemic stroke incidence escalating from 107 to 144 per 100,000 population between 1990 and 2019 (9). Although comprehensive national surveillance data for perioperative stroke remains limited, available multicenter investigations report incidence rates consistent with international patterns, typically ranging between 0.1% and 1% (10). Given China's substantial surgical volumes and rapidly aging population, the absolute number of affected elderly patients and their associated clinical outcomes warrant urgent attention and systematic investigation. If not addressed, this gap in perioperative stroke management can contribute to increased rates of postoperative disability, prolonged hospital stays, and elevated healthcare costs. These consequences not only reduce patients’ quality of life but also strain institutional resources, highlighting the urgency of early identification and effective prevention.

Against this epidemiological backdrop, the knowledge, attitudes, and practices (KAP) of healthcare professionals regarding perioperative ischemic stroke assume paramount clinical significance. Existing research has identified substantial deficiencies in clinicians’ comprehension of stroke pathophysiology, prognostic factors, and preventive strategies, alongside inadequate preoperative risk communication with patients (11, 12). The KAP survey methodology serves as a validated diagnostic instrument for assessing collective understanding, perceptions, and behaviors within specific populations regarding targeted clinical issues (13, 14). This approach proves particularly valuable in health literacy research, operating on the theoretical foundation that enhanced knowledge is positively associated with attitudes, which subsequently translate into improved clinical behaviors (15). Considering that optimal perioperative stroke management necessitates coordinated, proactive multidisciplinary intervention, the awareness levels and behavioral patterns of frontline healthcare providers represent critical determinants of risk reduction and outcome optimization.

Despite the evident clinical relevance of this issue, a substantial research gap persists in the systematic evaluation of healthcare workers’ KAP concerning perioperative ischemic stroke, particularly in the management of elderly surgical patients. Current literature remains limited in scope, frequently concentrating exclusively on anesthesiologists, employing restricted sample sizes, and demonstrating insufficient inclusion of other essential healthcare disciplines such as surgeons and nursing personnel (2, 12). Moreover, unlike most existing studies conducted in Western contexts, this investigation explores KAP within the Chinese healthcare setting, where institutional structures, training systems, and clinical pathways may differ significantly. This regional specificity helps to identify unique barriers and actionable targets that are not adequately addressed in existing international literature (12). To address this critical awareness gap, the present investigation examines the current KAP profile of healthcare professionals involved in elderly surgical patient care. Distinct from prior work, this study recruits a multidisciplinary sample rather than anesthesiologists alone, applies structural equation modeling (SEM) to characterize the interrelations among knowledge, attitude, and practice, and situates these findings within the Chinese regional healthcare context. In particular, this study aimed to (1) assess the KAP levels of healthcare professionals regarding perioperative ischemic stroke in elderly patients and (2) examine the relationships among these three dimensions, so as to inform the design of targeted, evidence-based educational strategies for this vulnerable patient population.

## Methods

### Study design and participants

A cross-sectional survey was conducted by Ningxia Hui Autonomous Region People's Hospital targeting healthcare professionals employed at the institution between 1 March 2025 and 1 June 2025. Participants were enrolled by convenience sampling; an anonymous questionnaire link was distributed to eligible staff through departmental work groups, and completion was voluntary. Anesthesiology staff constituted the largest group because the study was coordinated by the Department of Anesthesiology, and this overrepresentation is acknowledged as a potential source of selection bias in the Limitations. Ethical approval was obtained from the Ethics Committee of Ningxia Hui Autonomous Region People's Hospital (Approval No.: [2022]-LL-122), and informed consent was secured from all participants prior to data collection. The ethical approval remains valid for multiyear institutional research projects, and the study period (March–June 2025) fell within its approved time frame.

Eligible participants met the following inclusion criteria: (1) currently employed as healthcare professionals at the study hospital, (2) aged 18 years or older, (3) voluntarily agreed to participate and provided signed informed consent, and (4) demonstrated adequate cognitive function to comprehend and complete the questionnaire independently. Exclusion criteria included the following: (1) individuals who declined to participate or withdrew consent during the course of the study and (2) responses that were evidently incomplete or internally inconsistent, thereby affecting the validity of the data.

### Questionnaire

The questionnaire was developed with reference to existing literature and aligned with both national and international guidelines (16, 17) relevant to perioperative ischemic stroke. Following formulation of the initial draft, content and face validity were qualitatively evaluated by two subject matter specialists, who independently rated the relevance and clarity of each item. Their feedback informed subsequent revisions before questionnaire finalization. Formal quantitative content validity indices, including item-level content validity index (I-CVI) and scale-level content validity index (S-CVI), were not calculated. To evaluate the instrument's psychometric properties, a pilot test was administered to a sample of 40 healthcare professionals. The internal consistency of the questionnaire was acceptable, with an overall Cronbach's *α* coefficient of 0.952. Subscale coefficients were also satisfactory for knowledge (*α* = 0.951), attitude (*α* = 0.972), and practice (*α* = 0.911) domains. In the final sample of 273 respondents, the Cronbach's *α* coefficient was 0.907 for the overall questionnaire and 0.913, 0.937, and 0.894 for the knowledge, attitude, and practice domains, respectively.

The finalized version of the questionnaire, administered in Chinese, comprised four sections: demographic characteristics, knowledge, attitudes, and practices. The knowledge section consisted of 10 items, each rated on a three-point scale—responses were scored as 2 for “very familiar,” 1 for “heard of it,” and 0 for “unclear,” yielding a total possible score ranging from 0 to 20. The attitude section included eight items measured on a five-point Likert scale, with responses ranging from “strongly agree” (5) to “strongly disagree” (1), producing a cumulative score between 8 and 40. Similarly, the practice section comprised eight items, also assessed using a five-point scale from “always” (5) to “never” (1), resulting in a total score of 8–40. For example, a representative knowledge item was “Intraoperative risk factors to be monitored include hypotension, prolonged anesthesia, intraoperative blood loss, and temperature fluctuations” (K9), while a typical attitude item was “The use of a stroke risk scoring tool before surgery can guide clinical decision-making” (A3). For analytical purposes, a score equal to or exceeding 80% of the maximum possible score in each domain was considered indicative of adequate knowledge, positive attitudes, and proactive clinical practice. This proportional cut-off is consistent with the widely applied Bloom's cut-off criterion, in which ≥80% denotes a good/adequate level, and has been adopted in previous KAP studies using similar proportional thresholds (18, 19). It should be noted that the knowledge items assessed self-perceived familiarity rather than objectively tested competence; therefore, the knowledge scores reflect awareness rather than verified clinical knowledge.

### Questionnaire distribution and quality control

The questionnaire was set up on the “Wenjuanxing” platform, and an electronic questionnaire link was generated. To prevent duplicate submissions, the platform was configured to allow only one response per IP address and per device. All items were set as mandatory questions, so no missing data were generated for submitted questionnaires. All submissions were checked for completeness, consistency, and validity by members of the research team, and responses that were internally inconsistent or completed in an implausibly short time were excluded from analysis.

### Statistical analysis

Data analysis was conducted using SPSS version 27.0 (IBM Corp., Armonk, NY, USA) and AMOS version 26.0 (IBM Corp.). Continuous variables were described as means and standard deviations (SD). Categorical variables were expressed as frequencies and percentages (n, %). Continuous variables conforming to a normal distribution were compared using independent sample *t*-test or analysis of variance (ANOVA). Variables with skewed distribution were compared by the Wilcoxon–Mann–Whitney test or Kruskal–Wallis analysis of variance. Correlation analyses employed Pearson or Spearman correlation coefficients based on data distribution. To identify factors independently associated with proactive practice, multivariate logistic regression analysis was conducted. A score equal to or greater than 80% of the maximum value for the practice dimension was defined as the threshold for proactive practice. SEM was utilized to evaluate the statistical associations among KAP dimensions and baseline characteristics. The magnitude and significance of both direct and indirect effects were estimated. Model fit was evaluated using multiple indices, including the root mean square error of approximation (RMSEA), standardized root mean square residual (SRMR), Tucker–Lewis index (TLI), and comparative fit index (CFI). Acceptable model fit was defined by RMSEA and SRMR values below 0.08, and TLI and CFI values above 0.80. When the initial model did not meet these criteria, path analysis was subsequently applied to explore the mediating effects of key variables. All statistical tests were two-sided, and a *P*-value <0.05 was considered statistically significant. Construct validity of the questionnaire was examined by confirmatory factor analysis (CFA) using AMOS, and sampling adequacy was assessed with the Kaiser–Meyer–Olkin (KMO) measure.

## Results

### Baseline characteristics and KAP score

Initially, a total of 283 questionnaires were collected for this study. The following data were excluded: (1) one respondent did not agree with this study, (2) one questionnaire completed in less than 60 s; and (3) eight questionnaires showing identical responses across all items (Supplementary Figure S1). There were 273 remaining valid data cases, with an effective response rate of 96.47%. Among the 273 healthcare professionals (57.88% female; mean age 39.28 ± 9.42 years), the majority were physicians (75.46%) from anesthesiology departments (86.08%). Over half (53.85%) specialized in elderly patient care, and 53.85% received perioperative stroke training. Their knowledge, attitude, and practice scores were 14.41 ± 4.22, 36.77 ± 3.57, and 30.07 ± 6.51, respectively. Knowledge scores differed significantly by gender (males 15.21 ± 4.16 vs. females 13.82 ± 4.18), occupation (physicians 15.18 ± 3.92 vs. nurses 12.03 ± 4.27), education level (master's degree 15.53 ± 3.44 vs. bachelor's 14.05 ± 4.39), specialization in elderly care (specialized 15.16 ± 4.05 vs. non-specialized 13.57 ± 4.27), and training status (trained 15.48 ± 3.83 vs. untrained 13.16 ± 4.33). Attitude scores varied significantly with occupation (physicians 37.35 ± 3.16 vs. nurses 34.99 ± 4.16) and monthly income (lowest-income group 38.20 ± 2.54 vs. highest-income group 36.02 ± 3.95). Practice scores showed significant associations with income (lowest-income group 33.20 ± 5.97 vs. middle-income 29.01 ± 6.30), specialization in elderly care (specialized 31.01 ± 6.00 vs. non-specialized 29.01 ± 6.90), and training status (trained 31.56 ± 6.03 vs. untrained 28.33 ± 6.63) (all of *P* < 0.05) (Table 1).

**Table 1:** Demographic characteristics and KAP scores.

Characteristics	*N* (%)	Knowledge, mean ± SD	*P*	Attitude, mean ± SD	*P*	Practice, mean ± SD	*P*
*N* = 273							
Total score		14.41 ± 4.22		36.77 ± 3.57		30.07 ± 6.51	
Gender			0.003		0.422		0.564
Male	115						
(42.12%)	15.21 ± 4.16		37.08 ± 3.23		30.32 ± 6.33		
Female	158						
(57.88%)	13.82 ± 4.18		36.55 ± 3.80		29.88 ± 6.65		
Age	39.28 ± 9.42						
Occupation			<0.001		<0.001		0.977
Doctor	206 (75.46%)	15.18 ± 3.92		37.35 ± 3.16		30.05 ± 6.43	
Nurse	67 (24.54%)	12.03 ± 4.27		34.99 ± 4.16		30.10 ± 6.79	
Department			0.266		0.065		0.985
Surgery	24 (8.79%)	13.29 ± 4.54		35.04 ± 4.18		29.67 ± 6.15	
Intensive care unit (ICU)	3 (1.10%)	13.00 ± 3.61		33.33 ± 2.31		29.67 ± 5.13	
Anesthesiology	235 (86.08%)	14.61 ± 4.17		36.98 ± 3.47		30.14 ± 6.49	
Other	11 (4.03%)	12.82 ± 4.51		37.09 ± 3.48		29.55 ± 8.64	
Education level			0.026		0.935		0.374
Associate/Bachelor's degree	207 (75.82%)	14.05 ± 4.39		36.88 ± 3.68		29.90 ± 6.54	
Master's degree or above	66 (24.18%)	15.53 ± 3.44		37.08 ± 3.24		30.58 ± 6.45	
Years of work experience			0.093		0.340		0.521
<2	27 (9.89%)	14.63 ± 4.34		36.63 ± 3.54		30.41 ± 7.38	
2–5	41 (15.02%)	15.73 ± 3.35		37.61 ± 3.15		31.32 ± 5.42	
5–10	37 (13.55%)	13.35 ± 3.58		36.14 ± 4.07		29.68 ± 7.04	
>10	168 (61.54%)	14.28 ± 4.46		36.73 ± 3.55		29.79 ± 6.51	
Average weekly working hours			0.444		0.686		0.704
<40	8 (2.93%)	15.25 ± 3.85		36.50 ± 3.85		28.88 ± 6.45	
40–49	115 (42.12%)	14.87 ± 4.19		37.11 ± 3.51		30.25 ± 6.26	
50–59	91 (33.33%)	13.77 ± 4.45		36.57 ± 3.40		30.49 ± 6.77	
60–69	39 (14.29%)	14.18 ± 3.95		36.49 ± 3.87		29.44 ± 6.58	
>70	20 (7.33%)	14.75 ± 3.95		36.40 ± 4.17		28.75 ± 6.94	
Type of workplace			0.405		0.452		0.741
Public hospital	265 (97.07%)	14.37 ± 4.22		36.75 ± 3.59		30.10 ± 6.54	
Private hospital	8 (2.93%)	15.50 ± 4.44		37.63 ± 3.20		29.00 ± 5.63	
Hospital grade			0.399		0.672		0.577
Tertiary grade A	169 (61.90%)	14.65 ± 4.20		36.82 ± 3.66		30.43 ± 6.48	
Tertiary grade B	50 (18.32%)	14.38 ± 4.00		36.40 ± 3.46		30.30 ± 5.89	
Secondary grade A	47 (17.22%)	13.66 ± 4.59		36.68 ± 3.45		29.23 ± 7.08	
Other	7 (2.56%)	13.71 ± 3.86		38.86 ± 3.02		25.29 ± 6.55	
Monthly income per capita			0.184		0.021		0.041
<2,000	15 (5.49%)	16.07 ± 3.45		38.20 ± 2.54		33.20 ± 5.97	
2,000–5,000	98 (35.90%)	13.93 ± 4.22		36.14 ± 3.73		29.01 ± 6.30	
5,000–10,000	114 (41.76%)	14.75 ± 4.31		37.43 ± 3.25		30.78 ± 6.71	
>10,000	46 (16.85%)	14.02 ± 4.12		36.02 ± 3.95		29.52 ± 6.25	
Specializing in the care of elderly patients			0.002		0.936		0.010
Yes	144 (53.85%）	15.16 ± 4.05		36.73 ± 3.69		31.01 ± 6.00	
No	129 (47.25%)	13.57 ± 4.27		36.82 ± 3.45		29.01 ± 6.90	
Training related to perioperative ischemic stroke			<0.001		0.165		<0.001
Yes	147 (53.85%)	15.48 ± 3.83		36.99 ± 3.61		31.56 ± 6.03	
No	126 (46.15%)	13.16 ± 4.33		36.52 ± 3.53		28.33 ± 6.63	
Hospital with a dedicated geriatrics department			0.653		0.472		0.089
Yes	223 (81.68%)	14.49 ± 4.09		36.69 ± 3.63		30.44 ± 6.26	
No	50 (18.32%)	14.04 ± 4.80		37.14 ± 3.33		28.38 ± 7.36	

### Knowledge, attitude, and practice

The distribution of knowledge dimensions showed that the three questions with the highest number of participants choosing the “No understanding” option were as follows: “Intraoperative risk factors to be monitored include hypotension, prolonged anesthesia, intraoperative blood loss, and temperature fluctuations” (K9) with 65.2%, “Elderly patients should undergo comprehensive preoperative evaluation to identify and optimize modifiable risk factors, such as controlling blood pressure and blood glucose, and assessing cardiac function” (K7) with 61.5%, and “Elderly patients are a high-risk group for perioperative ischemic stroke, especially those with cardiovascular diseases or undergoing neurosurgical procedures” (K2) with 59.3% (Table 2).

**Table 2:** Distribution of knowledge dimension responses.

Items	*N* (%)		
	No understanding	Some understanding	Thorough understanding
1. Perioperative stroke is one of the serious postoperative complications in elderly surgical patients and often leads to a range of adverse clinical outcomes, including disability and even death.	150 (54.9%)	119 (43.6%)	4 (1.5%)
2. Elderly patients are a high-risk group for perioperative ischemic stroke, especially those with cardiovascular diseases or undergoing neurosurgical procedures.	162 (59.3%)	105 (38.5%)	6 (2.2%)
3. The peak incidence of ischemic stroke typically occurs within 24–72 h postoperatively, with significantly increased risks associated with cardiac surgery, carotid endarterectomy, and head and neck surgeries.	132 (48.4%)	127 (46.5%)	14 (5.1%)
4. The main causes of ischemic stroke include embolism, thrombosis, and reduced cerebral perfusion.	154 (56.4%)	115 (42.1%)	4 (1.5%)
5. Aspirin may increase perioperative bleeding in orthopedic surgeries, but it does not raise the incidence or mortality of bleeding-related complications.	133 (48.7%)	122 (44.7%)	18 (6.6%)
6. Are you aware of the high-risk factors for perioperative ischemic stroke, particularly in the elderly population (e.g., atrial fibrillation, atherosclerosis)?	142 (52.0%)	120 (44.0%)	11 (4.0%)
7. Elderly patients should undergo comprehensive preoperative evaluation to identify and optimize modifiable risk factors, such as controlling blood pressure and blood glucose, and assessing cardiac function.	168 (61.5%)	99 (36.3%)	6 (2.2%)
8. Preoperative assessment tools include the CHA_2_DS_2_-VASc score (for atrial fibrillation-related risk) and the NIHSS score (for assessing stroke severity).	56 (20.5%)	164 (60.1%)	53 (19.4%)
9. Intraoperative risk factors to be monitored include hypotension, prolonged anesthesia, intraoperative blood loss, and temperature fluctuations.	178 (65.2%)	90 (33.0%)	5 (1.8%)
10. Are you familiar with the indications and contraindications for thrombolytic therapy, as well as the clinical application of acute-phase pharmacologic treatments for perioperative ischemic stroke (e.g., tPA, antiplatelet agents, anticoagulation therapy)?	73 (26.7%)	176 (64.5%)	24 (8.8%)

NIHSS, National Institutes of Health Stroke Scale; tPA, tissue plasminogen activator

Participants’ attitudes were all very positive, with no respondents choosing negative options. There was relative room for improvement, with 4.4% being neutral about receiving a stroke risk assessment in the perioperative period (A1) and 4.0% being neutral toward the use of a stroke risk scoring tool to guide clinical decision-making before surgery (A3) (Table 3).

**Table 3:** Distribution of attitude dimension responses.

Items	*N* (%)				
	Strongly agree	Agree	Neutral	Disagree	Strongly disagree
1. You believe that all elderly patients should undergo stroke risk assessment during the perioperative period.	166 (60.8%)	95 (34.8%)	12 (4.4%)	0	0
2. You believe that the prevention of perioperative ischemic stroke (e.g., anticoagulation therapy, blood pressure management) is crucial for elderly patients.	180 (65.9%)	89 (32.6%)	4 (1.5%)	0	0
3. You support the use of stroke risk scoring tools (such as the CHA_2_DS_2_-VASc score) before surgery to guide clinical decision-making.	140 (51.3%)	122 (44.7%)	11 (4.0%)	0	0
4. You believe that the management of perioperative ischemic stroke requires multidisciplinary collaboration (e.g., neurology, anesthesiology, rehabilitation departments).	185 (67.8%)	82 (30.0%)	6 (2.2%)	0	0
5. You believe that the management of perioperative ischemic stroke should be individualized, with strategies adjusted based on patient history and intraoperative conditions.	184 (67.4%)	84 (30.8%)	5 (1.8%)	0	0
6. You believe that helping patients and their families understand risk prevention and management skills is an important responsibility of healthcare professionals in the context of perioperative stroke management.	168 (61.5%)	97 (35.5%)	8 (2.9%)	0	0
7. You are willing to proactively learn the latest guidelines on the diagnosis and treatment of perioperative ischemic stroke and apply them in clinical practice.	161 (59.0)	107 (39.2)	5 (1.8)	0	0
8. You believe it is very important to educate elderly patients and their families about the early symptoms of stroke and preventive measures.	173 (63.4)	97 (35.5)	3 (1.1)	0	0

Responses to the practice dimension showed that 17.6% rarely and 8.1% never adjusted the medication regimen based on the patient's postoperative condition (P4), 15.0% rarely and 7.0% never provided health education to perioperative stroke patients and their families on disease prevention and postoperative rehabilitation (P8), and 17.9% rarely and 6.6% never cooperated with the nursing team to ensure effective daily monitoring and rehabilitative care for perioperative stroke patients (P7) (Table 4).

**Table 4:** Distribution of practice dimension responses.

Items	*N* (%)				
	Always	Often	Occasionally	Rarely	Never
1. Do you closely monitor indicators such as blood pressure, blood glucose, and oxygen saturation during surgery?	215 (78.8)	51 (18.7%)	6 (2.2%)	1 (0.4%)	0
2. Do you actively propose or participate in discussions on intraoperative stroke risk prevention strategies within the surgical team?	88 (32.2%)	80 (29.3%)	80 (29.3%)	21 (7.7%)	4 (1.5%)
3. Postoperatively, do you closely monitor changes in the patient's neurological function (e.g., limb movement, speech ability)?	105 (38.5%)	109 (39.9%)	43 (15.8%)	14 (5.1%)	2 (0.7%)
4. Do you adjust the medication regimen (e.g., anticoagulation therapy, antiplatelet therapy) based on the patient's postoperative condition?	73 (26.7%)	76 (27.8%)	54 (19.8%)	48 (17.6%)	22 (8.1%)
5. Do you regularly collaborate with teams from departments such as neurology and anesthesiology to jointly manage patients with perioperative stroke?	75 (27.5%)	71 (26.0%)	78 (28.6%)	37 (13.6%)	12 (4.4%)
6. Do you participate in postoperative case discussions to analyze the causes of perioperative stroke and explore improvement measures?	55 (20.1%)	84 (30.8%)	76 (27.8%)	41 (15.0%)	17 (6.2%)
7. Do you cooperate with the nursing team to ensure that daily monitoring and rehabilitative care for perioperative stroke patients are effectively implemented?	60 (22.0%)	91 (33.3%)	55 (20.1%)	49 (17.9%)	18 (6.6%)
8. Do you provide health education to perioperative stroke patients and their families on disease prevention and postoperative rehabilitation?	60 (22.0%)	72 (26.4%)	81 (29.7%)	41 (15.0%)	19 (7.0%)

### Correlations between KAP

In the correlation analysis, significant positive correlations were found between knowledge and attitude (*r* = 0.386, *P* < 0.001), knowledge and practice (*r* = 0.300, *P* < 0.001), and attitude and practice (*r* = 0.272, *P* < 0.001), respectively (Table 5).

**Table 5:** Correlation analysis.

Dimensions	Knowledge	Attitude	Practice
Knowledge	1		
Attitude	0.386 (*P* < 0.001)	1	
Practice	0.300 (*P* < 0.001)	0.272 (*P* < 0.001)	1

### Univariate and multivariate analysis for practice

The cut-off value for the practice dimension was set at 80% of the total score, resulting in 113 participants (41.39%) falling below this threshold. Multivariate logistic regression showed that knowledge score (OR = 1.078, 95% CI: 1.004–1.157, *P* = 0.038), attitude score (OR = 1.155, 95% CI: 1.063–1.255, *P* < 0.001), and age (OR = 2.029, 95% CI: 1.079–3.817, *P* = 0.028) were independently associated with proactive practice (Table 6).

**Table 6:** Analysis of factors influencing good practice.

Characteristics	Univariate logistic regression		Multivariate logistic regression	
	OR (95% CI)	*P*	OR (95% CI)	*P*
Knowledge score	1.148 (1.078–1.223)	<0.001	1.078 (1.004–1.157)	0.038
Attitude score	1.192 (1.103–1.287)	<0.001	1.155 (1.063–1.255)	<0.001
Gender				
Male	1.396 (0.858–2.273)	0.180		
Female	Ref			
Age	0.999 (0.974–1.025)	0.939	2.029 (1.079–3.817)	0.028
Occupation			Ref	
Doctor	1.153 (0.656–2.026)	0.621		
Nurse	Ref			
Department				
Surgery	1.050 (0.239–4.615)	0.949		
ICU	0.875 (0.059–12.975)	0.923		
Anesthesiology	1.274 (0.363–4.471)	0.705		
Other	Ref			
Education level				
Associate/Bachelor's degree	0.741 (0.424–1.294)	0.292		
Master's degree or above	Ref			
Years of work experience			Ref	
<2	1.435 (0.635–3.245)	0.386		
2–5	1.472 (0.741–2.923)	0.270		
5–10	0.941 (0.452–1.958)	0.870		
>10	Ref			
Average weekly working hours				
<40	0.500 (0.080–3.127)	0.459		
40–49	0.930 (0.352–2.453)	0.883		
50–59	1.467 (0.548–3.927)	0.445		
60–69	0.840 (0.277–2.545)	0.758		
>70	Ref			
Type of workplace				
Public hospital	0.699 (0.171–2.854)	0.618		
Private hospital	Ref			
Hospital grade				
Tertiary grade A	4.674 (0.551–39.672)	0.158		
Tertiary grade B	4.000 (0.447–35.788)	0.215		
Secondary grade A	3.724 (0.414–33.516)	0.241		
Other	Ref			
Monthly income per capita				
<2,000	2.559 (0.775–8.444)	0.123		
2,000–5,000	0.827 (0.398–1.721)	0.612		
5,000–10,000	1.590 (0.788–3.210)	0.196		
>10,000	Ref			
Specializing in the care of elderly patients				
Yes	1.777 (1.090–2.899)	0.021	1.539 (0.901–2.628)	0.115
No	Ref		Ref	
Training related to perioperative ischemic stroke				
Yes	1.990 (1.215–3.260)	0.006	1.518 (0.882–2.611)	0.132
No	Ref		Ref	
Hospital with a dedicated geriatrics department				
Yes	1.467 (0.772–2.789)	0.242		
No	Ref			

Construct validity of the questionnaire was supported by confirmatory factor analysis. The KMO measure was 0.916 (*P* < 0.001), and the model showed acceptable fit (chi-square minimum divided by degrees of freedom (CMIN/DF) = 2.740, incremental fit index (IFI) = 0.894, TLI = 0.883, CFI = 0.894, and RMSEA = 0.080). Standardized factor loadings of the items on their respective knowledge, attitude, and practice constructs are presented in Figure 1.

**Figure 1:**
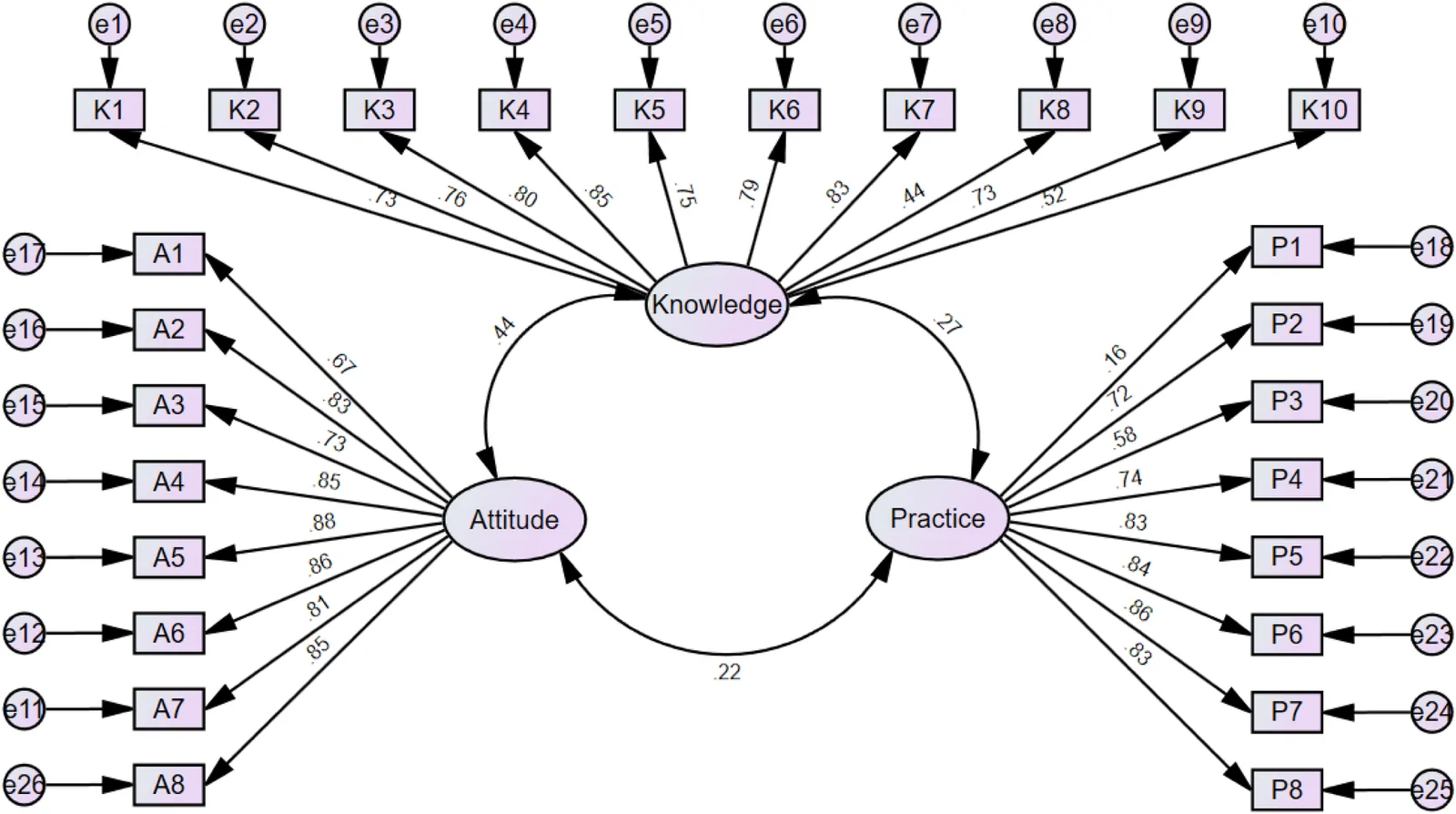
Confirmatory factor analysis of the KAP questionnaire (standardized estimates).

### SEM

The structural equation model demonstrated excellent fit: CMIN/DF = 2.100, IFI = 0.965, TLI = 0.908, and CFI = 0.963 (Supplementary Table S1). Significant direct associations were observed: Knowledge was positively associated with attitude (*β* = 0.352, *P* = 0.021) and practice (*β* = 0.203, *P* = 0.011), and attitude was positively associated with practice (*β* = 0.165, *P* = 0.034). Training related to perioperative ischemic stroke was negatively associated with knowledge (*β* = −0.287, *P* = 0.016) and practice (*β* = −0.181, *P* = 0.007), which may reflect limitations in the quality, relevance, or delivery of existing training programs. Specialization in elderly patient care was negatively associated with knowledge (*β* = −0.114, *P* = 0.039). Significant indirect effects showed that knowledge improved practice through attitude (*β* = 0.058, *P* = 0.023), training reduced attitude (*β* = −0.101, *P* = 0.008) and practice (*β* = −0.075, *P* = 0.008) via mediators, and specializing in elderly care lowered attitude (*β* = −0.040, *P* = 0.044) indirectly (Table 7 and Figure 2).

**Table 7:** Direct and indirect effects in the model.

Model paths	Standardized direct effects (95% CI)	*P*	Standardized indirect effects (95% CI)	*P*
Knowledge → Attitude	0.352 (0.227 to 0.438)	0.021		
Knowledge → Practice	0.203 (0.059 to 0.329)	0.011	0.058 (0.009 to 0.100)	0.023
Attitude → Practice	0.165 (0.010 to 0.256)	0.034		
Training related to perioperative ischemic stroke → Knowledge	−0.287 (−0.392 to −0.167)	0.016		
Training related to perioperative ischemic stroke → Attitude			−0.101 (−0.157 to −0.059)	0.008
Training related to perioperative ischemic stroke → Practice	−0.181 (−0.288 to −0.070)	0.007	−0.075 (−0.139 to −0.034)	0.008
Specializing in the care of elderly patients → Knowledge	−0.114 (−0.217 to 0.003)	0.039		
Specializing in the care of elderly patients → Attitude			−0.040 (−0.081 to −0.001)	0.044

**Figure 2:**
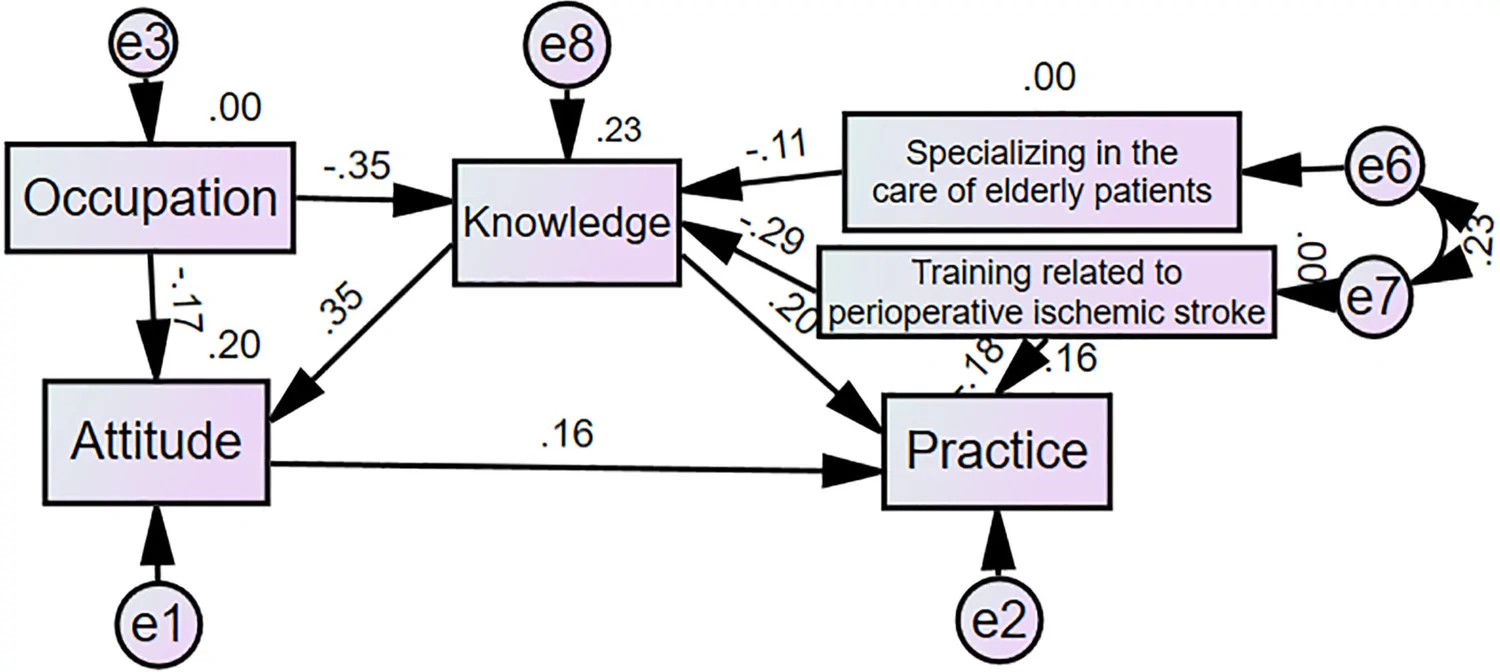
SEM.

## Discussion

Healthcare professionals demonstrated inadequate perceived knowledge, positive attitudes, and inactive practices toward perioperative ischemic stroke in elderly patients. This gap between attitude and practice highlights the potential value of targeted, evidence-based educational interventions that address knowledge awareness and practice-related barriers.

This study examined the KAP of healthcare professionals concerning perioperative ischemic stroke in elderly patients and revealed an observable discordance among the three dimensions. While most respondents expressed favorable attitudes toward stroke prevention and perioperative care, their reported levels of knowledge and practice fell short of the defined adequacy threshold. Related studies in perioperative risk management and stroke prevention have similarly highlighted a recurrent gap between conceptual endorsement and routine implementation, often mediated by variability in training quality, institutional expectations, and clinical workload (11, 20). Compared with similar studies conducted in Western countries, such as Canada and the United States, where structured stroke prevention protocols and interprofessional education are more established (2, 5), Chinese healthcare settings may lack standardized perioperative stroke pathways, which may account for the greater variability observed in practice behavior. For instance, Meng et al. reported that only a minority of Chinese anesthesiologists felt confident managing perioperative stroke, primarily due to inconsistent training and lack of institutional support (12). This contrasts with findings from developed healthcare systems, where multidisciplinary collaboration and guideline adherence are more routinely emphasized.

The positive correlations observed between knowledge, attitude, and practice support the foundational assumptions of the KAP framework. Knowledge was associated with more favorable attitudes and, independently, with more frequent engagement in recommended practices. However, the modest strength of these associations suggests that additional factors beyond cognitive awareness and attitudinal disposition may constrain the ability of practitioners to act in alignment with best practices. SEM offered a more integrated view of these interrelations, indicating that knowledge may be associated with behavior both directly and indirectly through attitudinal shifts. Although this pathway is consistent with conceptual models in health behavior research, the practical application remains sensitive to the specific context in which clinicians operate, particularly in complex perioperative settings (21, 22).

The analysis also identified age as a contributing factor, with older professionals more likely to report proactive practices. This may relate to cumulative clinical experience or a greater degree of autonomy in clinical decision-making, both of which can shape patterns of engagement over time. In contrast, the findings concerning training and specialization presented a more complex picture. Counter to expectations, those with prior training in perioperative stroke and those specializing in elderly care demonstrated lower knowledge and practice scores. While initially counterintuitive, such results are not without precedent. Previous evaluations of clinical training programs have found that educational content lacking clinical specificity or practical applicability may yield limited benefits and, in some cases, perpetuate misunderstandings or outdated concepts (23, 24). In the present study, this negative association may be attributed to several factors: The training received by participants could have been outdated, overly theoretical, or not tailored to real-world perioperative contexts. Moreover, the absence of standardized curricula and limited frequency or follow-up of such training sessions may reduce their effectiveness. If training is delivered in a one-size-fits-all format without practical integration or regular updates, it may reinforce superficial understanding rather than foster actionable competencies. Beyond these curricular explanations, alternative mechanisms merit consideration: (1) confounding by indication: Staff with perceived gaps or prior adverse events may be preferentially assigned to training, which links “training” with lower baseline knowledge or practice; (2) reverse causality: Clinicians who recognize deficits might self-select into training, so lower scores reflect their starting point rather than training effects; (3) exposure misclassification: Our binary “trained or untrained” variable aggregates heterogeneous formats, recency, duration, and competency assessment, which can dilute or invert associations; (4) knowledge decay: Lack of structured refreshers or opportunities for application may erode gains over time; and (5) system constraints: even adequate training may not translate into practice where workflow, team communication, and decision support are misaligned with stroke prevention behaviors. Taken together, the negative coefficient for training should be interpreted as hypothesis-generating rather than causal, pointing to the need to unpack who is trained, how they are trained, and whether the organizational environment enables transfer to practice. No information on the frequency, format, or recency of training was collected in this study; therefore, these explanations are speculative and highlight the need for future evaluation of training quality.

Improvement strategies should emphasize a shift from content-heavy lectures to competency-based, scenario-driven modules aligned with perioperative decision points (preoperative risk stratification, intraoperative hemodynamic management, and postoperative neurological monitoring), defining minimal proficiency with objective assessments, refreshing at regular intervals, and embedding audit and feedback and electronic decision support to reinforce behaviors at the bedside. Stratifying curricula by role (for example, anesthesiology, surgery, and nursing) and coupling training with explicit multidisciplinary pathways may further enhance translation into practice. Importantly, such educational modules should be developed on the basis of a documented appraisal of local practice gaps and measured adherence to perioperative stroke guidelines, ensuring that content is tailored to the specific behaviors that fall short rather than delivered generically. Future work should specifically compare different training modalities and recency, incorporate process measures such as adherence to monitoring targets and documentation, and explore trainees’ experiences qualitatively to clarify which components drive behavioral change.

With regard to the observed negative association between elderly care specialization and knowledge, this result should be interpreted with caution. In the present survey, “specialization in elderly care” referred to professionals working primarily in geriatric or chronic-disease wards, rather than those specifically trained in perioperative geriatric management. Their focus may center more on long-term functional or nursing care rather than perioperative stroke prevention, which could partially explain the lower knowledge scores observed. Future research should distinguish between general geriatric specialization and perioperative geriatric expertise to clarify this relationship.

A closer inspection of item-level responses revealed that awareness gaps clustered around areas requiring integration of multidisciplinary risk factors and application of preoperative assessment tools. Questions pertaining to intraoperative physiological monitoring and individualized risk stratification were among those with the lowest correct response rates. This trend points to the challenge of converting generalized awareness into specific competencies. Attitudinal responses were relatively uniform, indicating broad professional consensus on the importance of stroke prevention and team-based management. However, this consensus did not consistently manifest in reported behaviors. Participants frequently cited limited engagement in interprofessional coordination and educational outreach to patients, both of which are aspects critical to comprehensive perioperative stroke care. Observations from other studies suggest that such gaps may arise from the lack of structured institutional processes supporting collaborative practices, rather than from individual reluctance or disagreement with clinical priorities (25, 26).

Institutional and professional hierarchies may also shape how KAP dimensions evolve. Differences in scores across levels of education, income, and professional role suggest that access to clinical resources and opportunities for continuing education remain uneven (27, 28). These disparities likely contribute to the heterogeneity observed in knowledge and practice, particularly in settings where standardized stroke management protocols are either absent or inconsistently applied. The apparent disconnect between training participation and knowledge acquisition highlights the importance of ensuring not only the availability of educational interventions but also their content quality and contextual relevance.

Improving clinical practice in this area requires more than increasing exposure to training sessions. Interventions should be grounded in the clinical routines and decision points that healthcare professionals encounter. Embedding brief, scenario-based modules into existing work structures may allow for better integration of learning with daily tasks. Training should be tiered according to clinical responsibility and should reflect the interdisciplinary nature of stroke management. For instance, surgical and anesthesiology teams could benefit from case-based discussions focused on intraoperative risk scenarios, while nursing staff might be better supported through practical sessions on monitoring and patient education (29, 30). Content must be regularly updated in line with evolving guidelines and delivered in formats that allow for interactive engagement, rather than passive reception.

Institutional leadership plays a key role in enabling the application of new knowledge. This includes allocating time for staff participation in education, formalizing multidisciplinary communication pathways, and incorporating KAP assessments into existing quality improvement systems. The use of digital tools such as real-time clinical decision support systems may assist in reinforcing key behaviors during perioperative care, particularly when integrated with electronic health records (31).

This study has several limitations. First, its cross-sectional design limits the ability to establish causal relationships between knowledge, attitudes, and practices. Second, as the survey was conducted in a single hospital setting, the findings may not be generalizable to other institutions or healthcare systems. Because participants were recruited by convenience sampling and were drawn predominantly from a single specialty, selection bias cannot be excluded, and the sample may not be representative of the broader multidisciplinary workforce involved in perioperative care. Third, although the questionnaire underwent expert review for content and face validity and demonstrated satisfactory internal consistency and construct validity, formal quantitative content validity indices (I-CVI and S-CVI) were not calculated during questionnaire development. Therefore, the evidence supporting its content validity remains limited, and the findings should be interpreted with appropriate caution. Future studies should incorporate formal CVI assessment and more comprehensive psychometric validation. In addition, the exclusive reliance on self-reported questionnaire data introduces potential recall and reporting biases, as participants may have overestimated their attitudes or practices due to social desirability and subjective perception. These data do not directly reflect observed clinical behavior. Future studies should integrate self-reports with objective performance indicators or observational assessments to enhance measurement validity. Finally, detailed characteristics of the training received—such as its type, duration, format, and recency—were not collected, which limits the interpretation of the observed training-related associations. Future studies should capture these details to clarify the effect of training quality.

In conclusion, healthcare professionals demonstrated inadequate knowledge, positive attitudes, and inactive practices toward perioperative ischemic stroke in elderly patients. This misalignment between attitude and practice suggests that gaps in perceived knowledge and reported clinical practices coexist among healthcare professionals. Targeted educational interventions and capacity-building programs may be considered to address identified gaps in perceived knowledge and reported practices, although their effectiveness requires evaluation in future interventional studies.

## Data Availability

The original contributions presented in the study are included in the article/Supplementary Material; further inquiries can be directed to the corresponding author/s.
